# miR-124 and VAMP3 Act Antagonistically in Human Neuroblastoma

**DOI:** 10.3390/ijms241914877

**Published:** 2023-10-04

**Authors:** Xiaoxiao Zhang, Chengyong Yang, Zhen Meng, Huanhuan Zhong, Xutian Hou, Fenfen Wang, Yiping Lu, Jingjing Guo, Yan Zeng

**Affiliations:** 1 Department of Zoology, College of Life Sciences, Nanjing Agricultural University, Nanjing 210095, China; 2 Centre in Artificial Intelligence Driven Drug Discovery, Faculty of Applied Sciences, Macao Polytechnic University, Macao 999078, China

**Keywords:** miR-124, VAMP3, neuroblastoma, apoptosis, gene expression

## Abstract

Neuroblastoma (NB) is the most common extracranial solid tumor that affects developing nerve cells in the fetus, infants, and children. miR-124 is a microRNA (miRNA) enriched in neuronal tissues, and VAMP3 (vesicle-associated membrane protein 3) has been reported to be an miR-124 target, although the relationship between NB and miR-124 or VAMP3 is unknown. Our current work identified that miR-124 levels are high in NB cases and that elevated miR-124 correlates with worse NB outcomes. Conversely, depressed VAMP3 correlates with worse NB outcomes. To investigate the mechanisms by which miR-124 and VAMP3 regulate NB, we altered miR-124 or VAMP3 expression in human NB cells and observed that increased miR-124 and reduced VAMP3 stimulated cell proliferation and suppressed apoptosis, while increased VAMP3 had the opposite effects. Genome-wide mRNA expression analyses identified gene and pathway changes which might explain the NB cell phenotypes. Together, our studies suggest that miR-124 and VAMP3 could be potential new markers of NB and targets of NB treatments.

## 1. Introduction

NB is a malignant tumor composed of neuroblasts originating from the bone marrow, lymph nodes, or other organs, and is the most common solid tumor outside the cranium in children [1,2]. Treatments for NB include surgery, radiation therapy, and chemotherapy, and the success rates vary among individuals. According to the International Neuroblastoma Risk Group Staging System, NB can be classified into four categories: L1, L2, M, and MS, with a five-year survival rate below 50% in high-risk cases [1]. Therefore, it is imperative to study the mechanisms, diagnoses, and interventions involved in NB.

A wide range of genetic mutations have been identified in NB, including chromosomal variations, MYCN gene amplification, ALK gene mutations, LIN28B gene mutations, etc., although their precise roles in NB are still poorly understood [2,3,4]. Approximately 30% of patients have heterozygous deletions in the 1p36 chromosome region, and the severity of NB correlates with the level of deletions [5]. Since 1p36 contains hundreds of genes, however, a major question persists as to which gene or genes are responsible; currently, only one of the 1p36 genes, CHD5, has been extensively studied and potentially linked to NB [6,7,8]. Patients may also have multiple mutations, e.g., some NB cases have both 1p36 deletion and MYCN amplification, while some have 1p36 deletion or MYCN amplification, but not both [2,3].

miRNAs are a large class of approximately 22 nucleotide (nt)-long RNAs that are widely expressed in multicellular organisms and function mainly by binding to complementary sequences in the 3’ untranslated region (UTR) of target mRNAs to suppress gene expression, thereby regulating many biological processes and diseases [9,10,11,12,13,14]. miR-124-3p (miR-124 for short) is a conserved miRNA prominently expressed in neurons and believed to play an important role in regulating the development and function of the nervous system [15]. Altered miR-124 expression has been associated with certain cancers, although the mechanisms remain to be investigated [15,16]. It has been reported that inhibiting miR-124 induces cell differentiation and suppresses cell proliferation and apoptosis in human NB cells, and that such an effect can be overridden by expressing ELF4, an miR-124 target gene [17,18]. Other studies, however, observed that higher miR-124 induces NB cell differentiation or inhibits cell growth and promotes apoptosis variably [19,20,21]. Therefore, the function of miR-124 in NB is controversial.

VAMP3 is a member of the SNARE (soluble N-ethylmaleimide-sensitive factor attachment protein receptor) family of proteins that play crucial roles in intracellular and intercellular vesicle transport and membrane fusion [22,23,24,25,26,27]. In vitro experiments have shown that VAMP3 may influence ovarian cancer cell invasion and skin inflammation [28,29]. Additionally, gene expression data analysis suggests a potential association between VAMP3 and colon cancer and breast cancer [30,31]. In yeast, introduction of the human Bax gene induces cell apoptosis, while co-expression of human VAMP3 alleviates apoptosis [32]. This might be a mechanism by which VAMP3 impacts the growth of certain tumors. VAMP3 is also expressed in the central nervous system, suggesting its involvement in the release of neurotransmitters and participation in the learning and memory processes [22,33]. VAMP3 has been implicated as an miR-124 target in microglia [34]. Interestingly, the human VAMP3 gene resides in the 1p36 region often deleted in NB, although the relevance of VAMP3 to NB has not been directly examined. Therefore, in this study we employed bioinformatics and cell biology techniques to investigate the roles of miR-124 and VAMP3 in NB.

## 2. Results

### 2.1. Identification of High miR-124 and Low VAMP3 as Indicative of Poor Survival in NB

**Figure 1:**
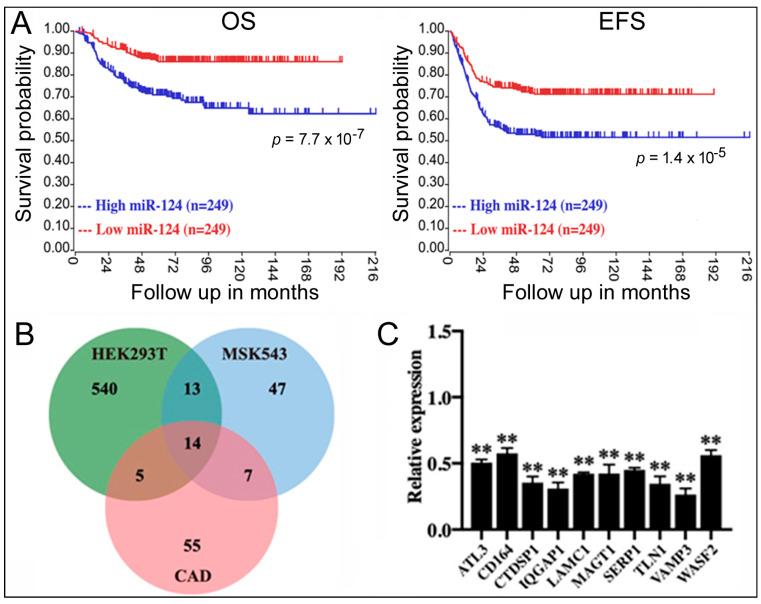
The role of miR-124 in NB and miR-124 target genes. (**A**) Kaplan–Meier analysis of OS and EFS in NB patients (GSE62564) based on miR-124 levels. *p* values are indicated. (**B**) Venn diagram showing differentially downregulated genes in cell lines overexpressing miR-124. (**C**) qPCR measurement of miR-124 target gene expression in 293T cells transfected with miR-124. Relative expression compares mRNA levels under the miR-124 transfection condition to those under the control RNA transfection (set at 1.0). Means and standard deviations are shown. **: *p* < 0.01.

To screen for the relationship between miRNAs and NB, we first examined the genome-wide miRNA qPCR data in the GSE121513 dataset, which contains eight embryonic stem cell lines, seven samples of normal fetal adrenal neuroblasts, seven samples of normal fetal adrenal cortex, and 95 NB samples. We found a significant difference in the miR-124 expression in NB samples, which showed a significant increase in miR-124 levels (average Ct = 19) compared to the embryonic stem cell lines (Ct > 35), normal fetal adrenal neuroblast samples (Ct > 35), and normal fetal adrenal cortex samples (Ct > 35). To explore this result further, we evaluated the survival data of 498 NB patients from the GSE62564 dataset; patients were divided based on the median miR-124 value into two groups: high expression (n = 249) and low expression (n = 249). Kaplan–Meier analysis demonstrated that high miR-124 expression was associated with a lower overall survival (OS) rate and event-free survival (EFS) rate, indicating a significant impact of miR-124 on NB patient outcomes (blue lines, Figure 1A).

miRNAs function by inhibiting target gene expression [9,10,11]. To identify the target genes of miR-124, we analyzed gene expression profiles before and after miR-124 overexpression in three cell types (293T/GSE18837, MSK543/GSE32876, and CAD/GSE8498). We identified differentially expressed genes in each dataset and found 14 genes that were inhibited by miR-124 in all three cell lines (Figure 1B). To validate the results, we transfected synthetic miR-124 into 293T cells and quantified the mRNA levels of those genes using qPCR. Increased miR-124 led to a significant downregulation of the mRNAs (relative expression < 1.0, displaying 10 of the mRNAs, Figure 1C), supporting their identities as miR-124 targets.

**Figure 2:**
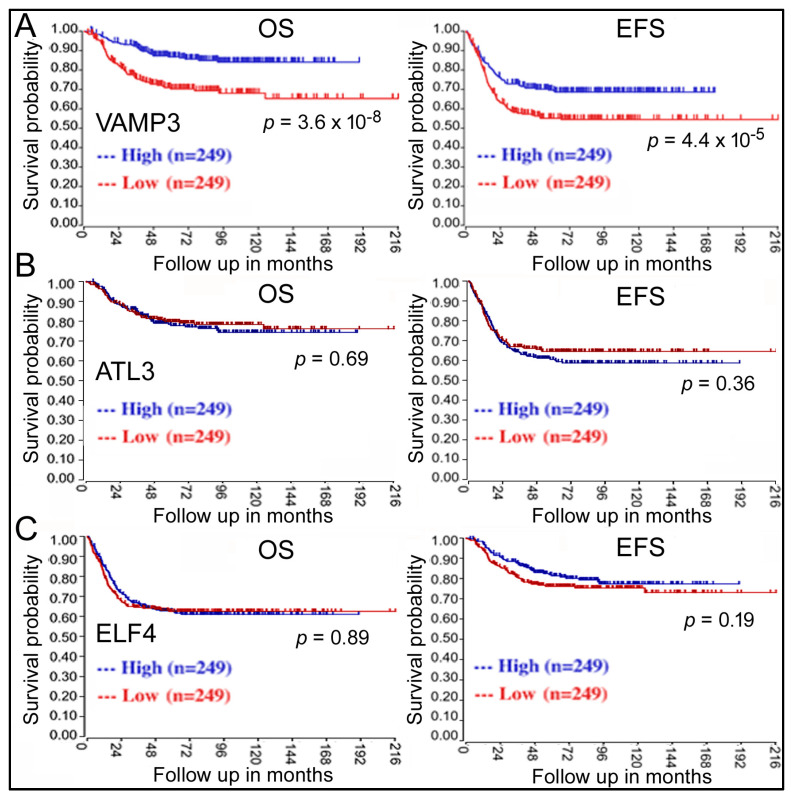
Kaplan–Meier analyses of the impacts of VAMP3 (**A**), ATL3 (**B**), and ELF4 (**C**) on NB patient survival in the GSE62564 dataset. Labeling is the same as in Figure 1A.

The next question is: do these genes play a role in NB? Kaplan–Meier analysis revealed that only VAMP3 displayed a significant correlation with NB, with low VAMP3 expression in NB samples being unfavorable for patient OS and EFS (red lines, Figure 2A). Other genes such as ATL3 (Figure 2B) did not show any effects. It has been reported that ELF4, another target gene of miR-124, regulates NB cells [18], but ELF4 expression was not associated with survival either (Figure 2C).

**Figure 3:**
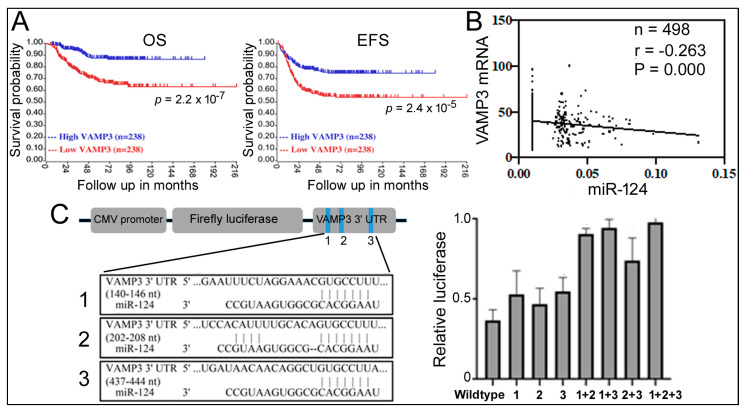
Relationship between VAMP3, miR-124, and NB. (**A**) Kaplan–Meier analysis of VAMP3 and NB in GSE45547. (**B**) Correlation between miR-124 and VAMP3 mRNA levels in NB patients from GSE62564. (**C**) Reporter gene assays examining miR-124 binding sites. The left panel is a schematic representation of the reporter gene construct, pCMV-luc-VAMP3, containing the 3’ UTR of VAMP3 mRNA along with the positions and sequences of potential miR-124 binding sites designated as 1, 2, and 3 (blue stripes in the graph). Mutations deleted the 3′ UTR sequences complementary to the miR-124 seed sequence (11). The right panel shows the relative luciferase expression (*y*-axis) with different 3’ UTR reporter genes on the *x*-axis. The reporters and an miR-124 or control RNA were co-transfected into 293T cells. Relative luciferase expression under the control RNA transfection condition was set as 1.0. Means and standard deviations are indicated on the graph.

Similar conclusions about VAMP3′s contribution were obtained from other NB survival datasets (GSE45547 [35], red lines for VAMP3, Figure 3A; and Table 1). Table 1 further analyzes additional genes in 1p36, such as CHD5, CDC42, MAD2L2, and FBXO6, as well as MYCN. The expression levels of CHD5, CDC42, MAD2L2, and FBXO6 are decreased in NB patients, with CHD5 already implicated in NB [6,7,8]. CDC42 is regulated by MYCN and is associated with the differentiation of NB cells [36,37]. MYCN is a gene located outside of 1p36 that is frequently amplified in NB, and some patients have both 1p36 deletion and MYCN amplification [2,3]. Table 1 shows persistent and significant CHD5, CDC42, and VAMP3 association with unfavorable NB outcomes at low gene expression. Table 1 list the results for MYCN as “high” for worse outcomes, consistent with MYCN amplification in NB. The MAD2L2 and FBXO6 results are inconsistent, while ELF4 exhibits no effects.

Due to the numerous mutations in NB and the probable overlaps between the mutations [2,3], we considered whether the impact of individual genes on NB can be explained by other mutations. To this end, we conducted a multivariable COX regression analysis in GSE62564 (there is no classification for 1p36 status in the available datasets, and only GSE62564 has miR-124 expression data). Table 2 presents the data for OS (EFS results were similar). ALK, LIN28B, and MYCN, known to be mutated in NB [2,3], showed a correlation with survival rate in the univariable analysis, but the *p* values were above 0.05 in the COX analysis. The same pattern was observed for VAMP3. This suggests that existing data might be insufficient to unravel the complex relationships between various mutations in NB. miR-124, however, showed significance in both the univariable and COX analyses, consistently highlighting the adverse impact of high miR-124 on survivals. Thus, we identified that the expression of miR-124 and VAMP3 can predict NB risk and progression, and the functions and mechanisms of miR-124 and VAMP3 merited further investigation.

### 2.2. Relationship between miR-124 and VAMP3

Since both miR-124 and VAMP3 influence NB, what is the relationship between miR-124 and VAMP3? Studies in microglia have reported VAMP3 as an miR-124 target [34], and the comparable effects of high miR-124 and low VAMP3 on NB (Figure 2 and Figure 3) support such a connection. To obtain more evidence, we performed a Pearson correlation analysis of miR-124 and VAMP3 expression levels in 498 NB patients from the GSE62564 dataset. We found a weak but significant and negative correlation between miR-124 and VAMP3 (Figure 3B), providing evidence that miR-124 inhibition of VAMP3 expression might be physiologically relevant.

The 3’ UTR of human VAMP3 mRNA contains three potential miR-124 recognition sites (Figure 3C), whose roles in miR-124 regulation were unknown [34]. To determine which site is necessary for miR-124 function, we constructed a luciferase reporter gene containing the whole 3′ UTR and then introduced a series of single, double, or triple mutations into these sites (Figure 3C). Co-transfection of an miR-124 mimic with the reporters in 293T cells showed that miR-124 was able to inhibit the expression of the wildtype reporter and all of its single-site mutations and that only double and triple mutations significantly reduced the responsiveness to miR-124 (Figure 3C). Thus, all three sites likely contribute to miR-124 regulation.

### 2.3. miR-124 and VAMP3 Expression Regulated the Cell Cycle and Apoptosis in NB Cells

**Figure 4:**
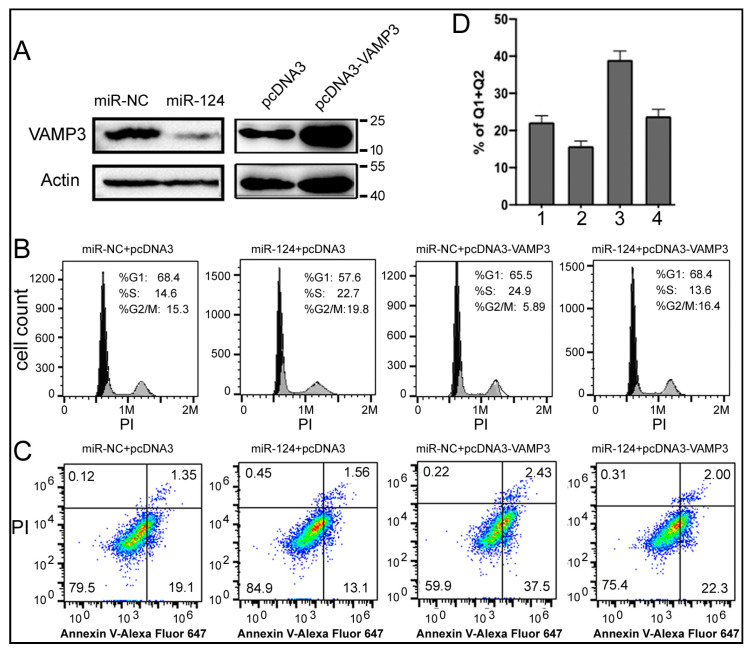
Effects of miR-124 and VAMP3 on the cell cycle and apoptosis. (**A**) Western blot analyses of VAMP3 and the control actin protein in SK-N-SH cells transfected with RNA or DNA as indicated. miR-NC: control RNA. Positions of protein markers in kilodaltons are indicated on the right. (**B**) Flow cytometry analysis of the cell cycle in SK-N-SH cells transfected with the indicated RNA and DNA. PI staining, indicative of the cellular DNA content, is on the *x*-axis. Percentages of cells in each phase of the cell cycle are listed in the graphs. (**C**) SK-N-SH cell apoptosis after transfection. The percentages of cells in the four quadrants are indicated in the graphs, with the right quadrants representing apoptotic cells. (**D**) Quantification of apoptosis in (**C**) by summing the percentages of cells in the right two quadrants. The means and standard deviations are shown. Samples 1, 2, 3, and 4 correspond to the samples from left to right in (**C**).

What are the mechanisms by which miR-124 and VAMP3 influence NB? To begin answering this question, we conducted experiments in the SK-N-SH human NB cell line. After transfecting SK-N-SH cells with the miR-124 mimic, the expression of VAMP3 protein was reduced (Figure 4A). We also constructed pcDNA3-VAMP3 plasmid-overexpressing human VAMP3 (Figure 4A). After confirming the ability to modulate miR-124 and VAMP3 levels, we performed cell staining and flow cytometry to evaluate the cell cycle and apoptosis phenotypes of SK-N-SH cells (Figure 4B–D).

Figure 4B shows that, compared to the negative control (miR-NC + pcDNA3), overexpressing miR-124 alone (miR-124 + pcDNA3) promoted cell proliferation, as indicated by an increase in cells in the G2/M phase from 15.3% to 19.8%. On the other hand, overexpressing VAMP3 alone (miR-NC + pcDNA3-VAMP3) elicited the opposite effect, resulting in a large decrease in cells in the G2/M phase from 15.3% to 5.89%. Co-transfection of miR-124 and pcDNA3-VAMP3 restored the cell cycle to a state similar to the negative control (16.4% vs. 15.3%). A simple explanation is that miR-124 promotes cell growth, while VAMP3 suppresses cell growth. Since the pcDNA3-VAMP3 plasmid lacks the 3’UTR of VAMP3, it should not be targeted by miR-124. Therefore, the results of co-transfection of miR-124 and pcDNA3-VAMP3 could be explained by other mechanisms (target genes) through which miR-124 promotes the cell cycle progression. Furthermore, the VAMP3 protein generated by pcDNA3-VAMP3 is only 2–3 times that of the endogenous level (Figure 4A), which may not be sufficient to significantly inhibit cell growth in the face of high miR-124. As for apoptosis, Figure 4C demonstrates that miR-124 inhibited apoptosis, while VAMP3 enhanced it, with the quantification plotted in Figure 4D. The differences between Samples 1 and 2, between 1 and 3, and between 2 and 3 are all significant (*p* < 0.05, Figure 4D). Thus, our results indicate that manipulating the levels of miR-124 and VAMP3 can alter the cell cycle and apoptosis profiles of NB cells, providing evidence and potential mechanisms for the impact of miR-124 and VAMP3 on NB patients.

### 2.4. Transcriptomic Analyses of the Effects of miR-124 and VAMP3

**Figure 5:**
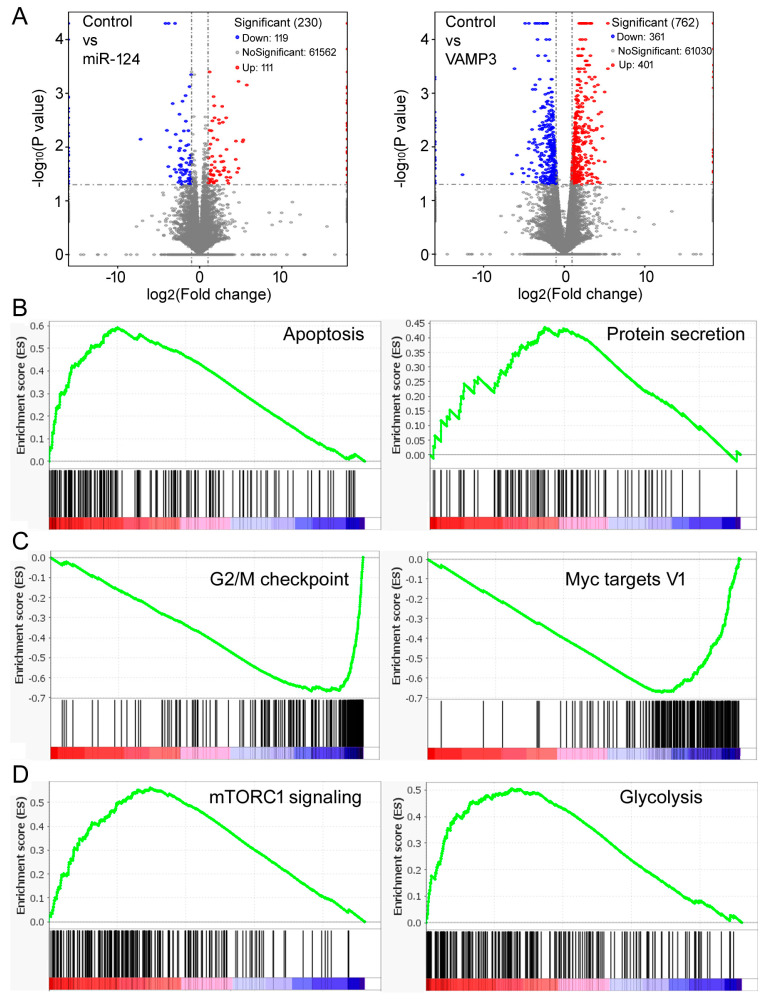
Gene expression and GSEA results. (**A**) Volcano plots comparing mRNA profiles of the control, miR-124-overexpressing, and VAMP3-overexpressing SK-N-SH cells. The numbers of genes in each category are listed in the graphs. (**B**) Representative gene sets enriched by high VAMP3 expression in the GSE62564 dataset. (**C**) Representative gene sets enriched by low VAMP3 expression. (**D**) Representative gene sets enriched by high miR-124 expression.

To further evaluate the molecular changes in NB cells, we performed mRNA-seq of SK-N-SH cells after miR-124 or VAMP3 transfection. Hundreds of differentially expressed genes were identified, from which potential KEGG pathways were inferred, including many involved in signal transduction and responses to viral infections (Supplementary Tables S1–S4). The miR-124 transfection resulted in fewer differentially expressed genes and fewer significant KEGG pathway changes (Figure 5A, left panel, Supplementary Tables S1 and S3) compared to VAMP3 (Figure 5A, right panel, Supplementary Tables S2 and S4). But the top ten pathways in the VAMP3 transfection sample (Supplementary Table S4) were all identified in the miR-124 sample (Supplementary Table S3), and eight of the top ten miR-124 pathways (Supplementary Table S3) were also present in VAMP3 (Supplementary Table S4), with one significant (ko05168, *p* < 0.05) in both, consistent with the functional relationship between miR-124 and VAMP3. These KEGG pathways are often associated with cell signaling and viral infections, probably relating to VAMP3′s role in membrane fusion and vesicle transport. Nonetheless, it was not immediately apparent how changes in such KEGG pathways would contribute to NB or explain the changes in SK-N-SH cell phenotypes, likely because of our small sample size. To gain further insights, we again turned to the existing, publicly available datasets and used gene set enrichment analysis (GSEA) [38] to compare global gene expression profiles and pathway changes under different miR-124 or VAMP3 levels.

We first analyzed the GSE62564 dataset. Table 3 shows the top gene sets with the FDR (false discovery rate) < 0.25 enriched in high VAMP3 and low VAMP3 samples, with the complete lists provided in Supplementary Tables S5 and S6. The “apoptosis” gene set was prominently enriched by high VAMP3 (Table 3, Figure 5B), supporting the result that VAMP3 enhanced apoptosis in SK-N-SH cells (Figure 4C,D). The “protein secretion” gene set was also enriched (Table 3, Figure 5B), consistent with VAMP3’s role as a SNARE protein. Other enriched gene sets, e.g., complement, PI3K AKT MTOR signaling, and p53 pathway, likewise hinted at VAMP3′s role in cancer or cellular transport. On the other hand, the “G2M checkpoint” gene set was enriched by low VAMP3 (Table 3, Figure 5C), consistent with the result that VAMP3 slowed cell division (Figure 4B). Low VAMP3 was also enriched in gene sets such as Myc targets, E2F targets, DNA repair, and mitotic spindle, which are known to be advantageous to the cell cycle progression (Table 3, Figure 5C, Supplementary Table S6).

We also tested miR-124 and found that essentially all gene sets were enriched in the high miR-124 samples (Table 4, Supplementary Table S7). Many have associations with tumor growth, such as mTORC1 signaling, glycolysis, hypoxia, and angiogenesis (Figure 5D, Table 4, Supplementary Table S7). These gene sets were enriched by both high and low VAMP3 (Table 3, Supplementary Tables S5 and S6), suggesting that the functions and mechanisms of miR-124 might be broader and more complex than those of VAMP3.

Next, we investigated dataset GSE45547 [35]. The results (Table 5, Supplementary Tables S8 and S9) were remarkably similar to those for GSE62564. High VAMP3 enriched gene sets such as protein secretion, apoptosis, and PI3K AKT MTOR signaling, while low VAMP3 enriched the same G2M checkpoint, Myc, and E2F targets gene sets. Lastly, we examined GSE16476 [39]. High VAMP3 had only one gene set, protein secretion, with an FDR < 0.25, but the same gene sets, such as apoptosis and PI3K AKT MTOR signaling, were also categorized in the high VAMP3 group (Supplementary Table S10). Low VAMP3 was enriched in G2M checkpoint, Myc, E2F targets, DNA repair, and mitotic spindle (FDR < 0.25, Supplementary Table S11). Together, these results point to potential mechanisms by which miR-124 and VAMP3 regulate NB at the molecular and cellular levels and contribute to NB patient survival.

## 3. Discussion

In this work, we used bioinformatics and cell biology approaches to screen for miRNAs and target genes involved in NB and then studied their mechanisms. The GSE62564 dataset contains both miR-124 and VAMP3 expression data from the same NB patients and tumor samples, allowing us to analyze the impact of miR-124 and VAMP3 on NB simultaneously. We showed elevated miR-124 and depressed VAMP3 levels correlated with worse OS and EFS in patients (Figure 1A and Figure 2A). The same VAMP3 result was replicated in additional datasets such as GSE45547 and GSE16476 (Figure 3A and Table 1), whereas other miR-124 targets such as ATL3 (Figure 1C) and ELF4 [18] displayed no such relationship to NB (Figure 2B,C, Table 1). The VAMP3 gene locus resides in the 1p36 chromosome region often deleted in NB, so we also examined how select individual 1p36 genes correlate with NB [5,6]. Lower expression of CHD5 and CDC42 was associated with worse NB outcomes (Table 1). Two other 1p36 genes, MAD2L2 and FBXO6, however, were not associated with NB (Table 1). Thus, not every gene in 1p36 or every miR-124 target influences NB, reinforcing the specificity of VAMP3 results here. On the other hand, only miR-124, not VAMP3 and a few well-known genes mutated in NB, was significant in COX analysis when examined together (Table 2), emphasizing miR-124′s unique role in NB and that the interplay between NB mutations merits further investigation. Indeed, NB patients harboring multiple mutations have been well documented, and these mutations may play overlapping or sequential roles during the process of tumorigenesis [2,3,4].

A previous report linked VAMP3 to miR-124 in microglia [34], although their detailed interaction and relationship to NB remained unknown. Our current work revealed that miR-124 repressed VAMP3 expression in multiple systems (Figure 1B,C), identified the 3′UTR sequences in VAMP3 mRNA responsible for miR-124 inhibition (Figure 3C), and showed miR-124 and VAMP3 levels negatively correlated with each other in NB patients (Figure 3B). These results firmly established VAMP3 as an miR-124 target and that such a regulation likely serves important biological functions, e.g., in NB.

By transient transfection and overexpression experiments we showed that increased miR-124 and increased VAMP3 had opposite effects on the growth and apoptosis of human NB cell line SK-N-SH (Figure 4B–D). This observation could explain why miR-124 and VAMP3 had different impacts on NB survivals, although future studies on additional phenotypes such as cell differentiation and more cell lines are needed to resolve the conflicting results [17,18,19,20,21]. RNA-seq analysis of the transfected SK-N-SH cells identified numerous changes in gene expression and KEGG pathways. In particular, VAMP3 might alter multiple signaling events at the membrane, perhaps by modifying membrane composition or morphology or transporting specific cargoes (Supplementary Table S4). Nevertheless, it is not straightforward to pinpoint, likely as a result of our small sample size, how these changes lead to the SK-N-SH phenotypes, and NB. Consequently, we again turned to public datasets GSE62564, GSE45547, and GSE16476 by GSEA. All three datasets yielded very similar results, as the nearly identical gene sets were enriched by high VAMP3, while other, nearly identical gene sets were enriched by low VAMP3 (Table 3, Table 4 and Table 5, Supplementary Tables S5–S11). The enrichment was consistent with increased VAMP3 suppressing apoptosis and with increased miR-124 and, hence, decreased VAMP3, stimulating cell proliferation. The fact that all three datasets yielded the same gene sets is significant, as they examined distinct NB populations at different times using diverse high-throughput platforms [35,39,40]. These results suggest that the gene sets and mechanisms we identified are likely universal and critical for the etiology and progression of NB. Based on results presented here, increasing or maintaining VAMP3 expression could be a new NB treatment strategy.

As for gene sets enriched by high miR-124 in GSE62564, they encompassed those enriched by high and low VAMP3 (Table 4, Supplementary Table S7), suggesting that miR-124 has broader, more complex mechanisms and that targets other than VAMP3 might also regulate NB. This is consistent with the results in Figure 4, where increased miR-124 was able to suppress the effects of an ectopically expressed VAMP3 even though the latter was not a target of miR-124 inhibition.

In summary, our data showed that miR-124 and VAMP3 act antagonistically and are potentially new biomarkers and therapeutic targets in NB. While Kaplan–Meier survival analysis and GSEA have painted a very consistent picture of how miR-124 and especially VAMP3 could impact NB, it is still correlative evidence. For example, VAMP3 might happen to be just a passenger to another gene(s) driving NB progression (Table 2). Thus, much work is needed to unravel the mechanisms of miR-124 and VAMP3 in more detail, e.g., in other NB cell lines and animal models, molecular changes in the cell cycle and apoptosis induced by miR-124 and VAMP3, additional miR-124 targets, and potential VAMP3 cargoes involved in NB.

## 4. Materials and Methods

### 4.1. Molecular Cloning

Restriction enzymes, PCR enzymes, and T4 DNA ligase were obtained from New England BioLabs (Ipswich, MA, USA). pRL-CMV, the control, and Renilla luciferase-expressing plasmid were from Promega (Madison, WI, USA). pCMV-luc has been described [41]. To test the interaction between miR-124 and VAMP3, the VAMP3 3′UTR sequence was amplified from a human cDNA library [42] using primers 5′-GCGCTAGCGAACCAGCGGAACTCAAAAC-3′ and 5′-GCCTCGAGAAGCTTTGGTCATAGCC-3′ and the PCR product digested with NheI and XhoI was inserted into the NheI and XhoI sites of pCMV-luc to make pCMV-luc-VAMP3. Mutational variations of the reporter were constructed using the Quikchange method (Stratagene, La Jolla, CA, USA) with primer sets 5′-GTTGAATTTCTAGGAAACTGTTTTTTAATATGCACT-3′, 5′-AGTGCATATTAAAAAACAGTTTCCTAGAAATTCAAC-3′, 5′-CCGTCCACATTTTGCACATACAGATTTACGTATGGG-3′, 5′-CCCATACGTAAATCTGTATGTGCAAAATGTGGACGG-3′, 5′-CTGTGATAACAACAGGCTTTTTGATAATTTTCTGAT-3′, and 5′-ATCAGAAAATTATCAAAAAGCCTGTTGTTATCACAG-3′. Sanger sequencing (Sangon, Shanghai, China) verified the identities of the clones. The VAMP3-overexpressing plasmid was constructed by amplifying the VAMP3 coding sequence using primers 5′-GCAAGCTTATGTCTACAGGTCCAAC-3′ and 5′-GCCTCGAGTGAAGAGACAACCCAC-3′, digesting the PCR product with HindIII and XhoI, and inserting it into the HindIII and XhoI sites of pcDNA3 to make pcDNA3-VAMP3 (Invitrogen, Waltham, MA, USA).

### 4.2. Cell Culture and Transfection

The human 293T and SK-N-SH cell lines were acquired from the Shanghai Institute of Biochemistry and Cell Biology of the Chinese Academy of Sciences (Shanghai, China). 293T cells were cultured in the Dulbecco’s modified Eagle’s medium supplemented with 10% fetal bovine serum and 2 mM L-glutamine, while SK-N-SH cells were cultured in Minimum Essential Medium containing 10% fetal bovine serum and 2 mM L-glutamine (Invitrogen). Both cell lines were maintained at 37 °C with 5% CO_2_ in a cell culture incubator. Cells were transfected using Lipofectamine 2000 (Invitrogen). For 293T transfection, cells were typically seeded in a 24-well plate and transfected with 400 ng of an miR-124 mimic or a control RNA (Sangon) and additional plasmid DNAs if needed; then, gene expression was examined two days later. For SK-N-SH transfection, cells were seeded in a six-well plate and transfected with 1 µg of pcDNA3 or pcDNA3-VAMP3 and 1 µg of miR-124 or a control RNA, then assayed 48–72 h later.

### 4.3. RNA Isolation and Real-Time PCR (qPCR) Analyses

Total RNA was isolated from 293T and SK-N-SH cells using Trizol (Invitrogen). SuperScript III First-Strand Synthesis SuperMix (Invitrogen) was used to reverse transcribe 293T RNA to produce cDNA. qPCR reactions were carried out on the QuantStudio 6 Flex Real-Time PCR System (Thermo Fisher Scientific, Waltham, MA, USA) using the SYBR Green qPCR Master Mix (Thermo Fisher Scientific). The relative expression of target genes was calculated with the 2^−ΔΔCT^ method normalized to that of the β-Actin mRNA. qPCR primers were synthesized by Sangon: Actin: 5′-GGACTTCGAGCAAGAGATGG-3′ and 5′-AGCACTGTGTTGGCGTACAG-3′; ATL3: 5′-TGGACTCCAAGGAGGAATGGCA-3′ and 5′-GAGAAAGCAGGTGACATCGGAG-3′; CD164: 5′-AACGTGACGACTTTAGCGCCCA-3′ and 5′-ACGCAGCTGTTTCGACCTTCAC-3′; CTDSP1: 5′-CTGCCTCCTATGTCTTCCATCC-3′ and 5′-ACGGCTGAGTTGCTCGAAGAAG-3′; IQGAP1: 5′-CCGTGGATACTTAGTTCGACAGG-3′ and 5′-AGCGCAGGTAAGCTAACCGATC-3′; LAMC1: 5′-CTGTGAGGTCAACCACTTTGGG-3′ and 5′-AGCCTTCTCTGCATTCACAGCG-3′; MAGT1: 5′-CGTCATGTTCACTGCTCTCCAAC-3′ and 5′-CCTGTTGGTGAATGCACTGGAG-3′; SERP1: 5′-GCAACGTCGCCAAGACCTC-3′ and 5′-CACATGCCCATCCTGATACTTTG-3′; TLN1: 5′-TTGGAGATGCCAGCAAGCGACT-3′ and 5′-CCAGTTCTGTGGCTGCCTGATT-3′; VAMP3: 5′-GCTCTCTGAGTTAGACGACCGT-3′ and 5′-CCAGAACAGTAATCCCGATTGCC-3′; and WASF2: 5′-CACCACAGTCAGACTCTGCTTC-3′ and 5′-CCAGATCCTCTTTGGTTGTCCAC-3′.

### 4.4. Protein Analyses

Cell lysis, protein extraction, and Western blotting were performed as described [43]. The antibody against β-Actin was from Proteintech (Rosemont, IL, USA), and the antibody against VAMP3 was from Sangon. For reporter assays, 293T cells were seeded in a 24-well plate and transfected with 20 ng pCMV-luc-VAMP3, 5 ng pRL-CMV, and 400 ng of an miR-124 mimic or a control RNA, and luciferase activities were measured using the Dual-Luciferase Assay System (Promega) two days later.

### 4.5. The Cell Cycle and Apoptosis Analyses

All cell culture experiments were conducted at least three times to ensure consistent results. The Cell Cycle and Apoptosis Analysis Kit (YEASEN, Shanghai, China) was used to study the cell cycle of SK-N-SH cells after transfection. Approximately 2 × 10^5^ cells were washed and collected by centrifugation at 1000× *g* for 5 min. The cells were fixed in cold 70% ethanol, washed, and stained with a propidium iodide (PI) solution containing RNase A. The stained cells were filtered through a 400-mesh sieve and analyzed using a BD Accuri C6 flow cytometer and the FlowJo v10.6 software (Becton Dickinson, Franklin Lakes, NJ, USA). The Annexin V-Alexa Fluor 647/PI Apoptosis Detection Kit (YEASEN) was used to study the apoptosis of SK-N-SH cells after transfection. Harvested cells were stained with Annexin V-Alexa Fluor 647 and PI and similarly analyzed using a BD Accuri C6 flow cytometer and the FlowJo v10.6 software.

### 4.6. High-Throughput RNA Sequencing (RNA-seq) and Data Analyses

For RNA-seq (Azenta Life Sciences, Burlington, MA, USA), a cDNA library was prepared from 1 µg of SK-N-SH total RNA. Briefly, the poly(A) mRNA was isolated using oligo(dT) beads and fragmented with divalent cations at high temperature. Primed with random primers, the first-strand cDNA and then the second-strand cDNA were synthesized. The double-stranded cDNA was tailed with dA, followed by a T-A ligation to add adaptors to both ends, which was amplified by PCR using adaptor primers and then loaded onto an Illumina HiSeq/Illumina Novaseq/MGI2000 instrument for sequencing using a 2 × 150 paired-end (PE) configuration according to the manufacturer’s instructions (Illumina, San Diego, CA, USA). Raw sequence data were processed by Cutadapt (V1.9.1) to acquire high quality clean data, which were then aligned to the human reference genome GRCh38 with Hisat2 (v2.0.1). HTSeq (v0.6.1) was used to obtain gene expression levels from the pair-end clean data. RNA-seq data have been deposited in the Gene Expression Omnibus under the accession number GSE240277. The DESeq2 Bioconductor package was used to identified differential gene expression, from which sample differences in KEGG (Kyoto Encyclopedia of Genes and Genomes) pathways were deducted.

### 4.7. Bioinformatics and Statistics Analyses

Datasets GSE121513, GSE62564, GSE18837, GSE32876, GSE8498, GSE45547, and GSE16476 were downloaded from https://www.ncbi.nlm.nih.gov/geo/ (accessed on 10 December 2019). Kaplan–Meier survival analysis was performed on human NB patients using the R2 genomics analysis and visualization platform (http://hgserver1.amc.nl/cgi-bin/r2/main.cgi (accessed on 10 December 2019)). In brief, dataset samples were categorized based on the expression levels of individual genes and divided into high and low expression groups using the median expression values. The log-rank test compared differences between the groups, with a significance threshold set at *p* < 0.05.

GSEA was applied to publicly available datasets. Dataset samples were categorized based on the expression levels of a specific gene such as VAMP3 and divided into high and low expression groups. GSEA was performed using the MSigDB dataset h.all.v2023.1.Hs.symbols.gmt, and the significance of enrichment scores was evaluated through 1000 permutations.

GraphPad Prism (GraphPad Software, San Diego, CA, USA) and SPSS (IBM, Armonk, NY, USA) were used for the Pearson correlation test and univariable and COX regression analyses.

## Figures and Tables

**Table 1:** Kaplan–Meier analyses of the relationship between gene expression and OS or EFS in the GSE62564, GSE45547, and GSE16476 NB datasets. Each dataset is divided into two groups based on individual gene expression levels, with numbers in parentheses denoting sample sizes and *p* values listed in the table. For *p* < 0.05, low expression is assumed to be associated with poorer outcomes; otherwise, “high” is indicated for high expression associated with adverse effects.

Genes	GSE62564 (249 vs. 249)		GSE45547 (238 vs. 238)		GSE16476 (44 vs. 44)	
	OS	EFS	OS	EFS	OS	EFS
CHD5	3.80 × 10^−24^	8.70 × 10^−22^	3.50 × 10^−20^	1.50 × 10^−18^	2.10 × 10^−6^	9.40 × 10^−6^
CDC42	1.50 × 10^−4^	0.013	8.40 × 10^−4^	0.036	6.70 × 10^−6^	2.50 × 10^−6^
FBXO6	1.30 × 10^−6^	2.50 × 10^−4^	0.023	0.36	0.87	0.44
MAD2L2	9.60 × 10^−4^	4.10 × 10^−3^	0.054	0.20	0.054	0.21
VAMP3	3.60 × 10^−8^	4.40 × 10^−5^	2.20 × 10^−7^	2.40 × 10^−5^	0.038	0.012
MYCN	high, 6.9 × 10^−3^	0.06	high, 0.0031	high, 0.010	0.054	high, 0.018
ELF4	0.19	0.89	1	0.46	0.15	high, 0.018

**Table 2:** Univariable and COX regression analyses based on GSE62564. HR, hazard ratio; CI, confidence interval.

Parameter	Univariable			COX		
	HR	95% CI	*p*	HR	95% CI	*p*
Age	1	1.00–1.00	<0.001	1	1.00–1.00	<0.001
High risk	21.01	11.70–37.74	<0.001	6.89	3.33–14.26	<0.001
NB stage	1.76	1.49–2.07	<0.001	1.39	1.06–1.81	0.017
ALK	1.55	1.37–1.75	<0.001	1.14	0.99–1.31	0.052
LIN28B	1.75	1.45–2.10	<0.001	1.15	0.92–1.45	0.227
MYCN	1.53	1.40–1.67	<0.001	1.2	0.93–1.55	0.165
VAMP3	0.37	0.28–0.48	<0.001	1.53	0.97–2.43	0.069
miR-124	1.35	1.12–1.63	0.001	1.37	1.13–1.65	0.001

**Table 3:** GSEA results of MSigDB gene sets enriched in high vs. low VAMP3 expression samples in GSE62564. Top ranked gene sets with an FDR < 0.25 are shown here (see text for details). Size: number of genes in the gene sets with detectable mRNA expression. ES: enrichment score. NES: normalized ES. *p*-val: nominal *p* values.

Gene Sets	Size	ES	NES	*p*-val	FDR
GSE62564: high VAMP3					
APOPTOSIS	161	0.59	1.80	0.000	0.107
PROTEIN_SECRETION	96	0.44	1.79	0.033	0.065
COMPLEMENT	200	0.63	1.72	0.000	0.104
IL2_STAT5_SIGNALING	199	0.60	1.68	0.006	0.127
APICAL_JUNCTION	200	0.52	1.66	0.006	0.127
HEME_METABOLISM	200	0.43	1.64	0.014	0.133
PI3K_AKT_MTOR_SIGNALING	104	0.40	1.62	0.025	0.133
UV_RESPONSE_DN	144	0.52	1.62	0.025	0.133
P53_PATHWAY	200	0.50	1.60	0.022	0.121
MYOGENESIS	200	0.54	1.59	0.050	0.118
GSE62564: low VAMP3					
MYC_TARGETS_V1	200	−0.67	−1.72	0.016	0.092
MYC_TARGETS_V2	58	−0.70	−1.70	0.016	0.055
E2F_TARGETS	200	−0.72	−1.62	0.038	0.090
G2M_CHECKPOINT	200	−0.67	−1.59	0.065	0.084

**Table 4:** GSEA results of MSigDB gene sets enriched in high miR-124 expression samples in GSE62564. The top 10 gene sets are shown here, and the complete GSEA list is provided in Supplementary Table S7. See Table 3 for abbreviations.

Gene Sets	Size	ES	NES	*p*-val	FDR
GSE62564: high miR-124					
CHOLESTEROL_HOMEOSTASIS	74	0.64	1.93	0.000	0.014
MTORC1_SIGNALING	200	0.56	1.91	0.016	0.015
GLYCOLYSIS	200	0.50	1.89	0.000	0.012
EPITHELIAL_MESENCHYMAL_TRANSITION	200	0.72	1.81	0.000	0.034
HYPOXIA	200	0.56	1.77	0.006	0.043
ESTROGEN_RESPONSE_LATE	200	0.55	1.77	0.000	0.036
UV_RESPONSE_UP	158	0.46	1.74	0.006	0.041
P53_PATHWAY	200	0.57	1.77	0.020	0.044
ANGIOGENESIS	36	0.70	1.69	0.028	0.058
ADIPOGENESIS	199	0.46	1.68	0.021	0.057

**Table 5:** GSEA results of MSigDB gene sets enriched in high vs. low VAMP3 expression samples in GSE45547. Top gene sets with an FDR < 0.25 are listed here, and complete GSEA lists are provided in Supplementary Tables S8 and S9. For abbreviations, see Table 3.

Gene Sets	Size	ES	NES	*p*-val	FDR
GSE45547: high VAMP3					
PROTEIN_SECRETION	91	0.65	2.07	0.000	0.019
APOPTOSIS	155	0.50	1.82	0.010	0.163
UV_RESPONSE_DN	133	0.51	1.82	0.010	0.114
COMPLEMENT	193	0.51	1.78	0.013	0.113
HEDGEHOG_SIGNALING	35	0.51	1.72	0.004	0.139
TNFA_SIGNALING_VIA_NFKB	187	0.58	1.72	0.050	0.116
PI3K_AKT_MTOR_SIGNALING	101	0.43	1.68	0.010	0.132
KRAS_SIGNALING_UP	190	0.44	1.64	0.025	0.155
INTERFERON_GAMMA_RESPONSE	190	0.56	1.58	0.111	0.204
HEME_METABOLISM	183	0.37	1.57	0.006	0.184
GSE45547: low VAMP3					
MYC_TARGETS_V2	51	−0.74	−2.03	0.000	0.005
G2M_CHECKPOINT	178	−0.54	−1.58	0.102	0.173
MYC_TARGETS_V1	179	−0.51	−1.46	0.123	0.213
E2F_TARGETS	179	−0.55	−1.45	0.166	0.166

## Data Availability

The data presented in this study are available in Supplementary Tables S1–S11, and datasets GSE121513, GSE62564, GSE18837, GSE32876, GSE8498, GSE45547, GSE16476, and GSE240277 from https://www.ncbi.nlm.nih.gov/geo/ (accessed on 10 December 2019).

## Supplementary Materials

The following supporting information can be downloaded at: https://www.mdpi.com/article/10.3390/ijms241914877/s1.
